# Attachment and parental bond: impact on psychopathology, mental health and quality of life of hemodialysis patients: a cross-sectional study

**DOI:** 10.1186/s40359-023-01246-8

**Published:** 2023-07-15

**Authors:** Concetta De Pasquale, Maria Luisa Pistorio, Massimiliano Veroux, Gabriella Sapienza, Alberto Florio, Zira Hichy, Burcin Ekser, Alessia Giaquinta, Pierfrancesco Veroux

**Affiliations:** 1 Vascular Surgery and Organ Transplant Unit, Department of Educational Science, University of Catania, Catania, Italy; 2 Vascular Surgery and Organ Transplant Unit, Department of General Surgery and Medical-Surgical Specialties, University Hospital of Catania, Via Santa Sofia, 84, 95123 Catania, Italy; 3 Organ Transplant Unit, Department of Surgical and Medical Sciences and Advanced Technologies, University Hospital of Catania, Catania, Italy; 4 Department of Educational Sciences, University of Catania, Catania, Italy; 5 Department of Surgery, Indiana University School of Medicine, Indianapolis, USA

**Keywords:** Attachment, Parental bond, Psychopathology, Hemodialysis, Quality of life, Mental health

## Abstract

**Background:**

Attachment theory represents a reference model for understanding better how pre-existing personality factors can influence the coping with some chronic conditions. The onset of a chronic disease can represent a "threat" to the relationships between the subject and parental figures according to the type of bond that already exists. The aim of our study was to explore attachment styles in a sample of hemodialysis patients, hypothesizing that a secure attachment bond can constitute a protective factor for the quality of life and mental health in this type of patients.

**Design:**

We used a cross-sectional design.

**Methods:**

Fifty hemodialysis patients were given the following tests: Attachment Style Questionnaire (ASQ) to assess attachment styles, Parental Bonding Instrument (PBI) to assess parental bonding, Short Form Health Survey-36 (SF-36) for perceived quality of life and Middlesex Hospital Questionnaire (MHQ) to detect key psychological symptoms and relevant traits.

**Results:**

The results showed that secure attachment style correlated with good general health (*r* = 0.339; p < 0.05), good mental health (*r* = 0.547; *p* < 0.001) and mental component scale (*r* = 0.373; *p* < 0.05) of SF-36. Secure attachment was also significantly associated with mental health (B = 1.104; *p* = .002) of the SF-36.

**Conclusions:**

The results confirmed the positive role of a secure attachment style for adequate psychological health. Early identification of patients with dysfunctional attachment styles will make it possible to offer them targeted interventions to improve their ability to accept, adapt and manage the disease and to maintain adequate psychological well-being.

## Introduction

Many studies have shown a significant relationship between health conditions and attachment [1–5]. Attachment theory represents a reference model that allows us to better understand how some pre-existing personality factors can influence the coping with some chronic conditions [6–8]. John Bowlby believed that attachment was of the secure type, when the child feels he has protection, a sense of security, affection from the reference figure; of insecure type when the child in the relationship with the attachment figure prevail instability, excessive prudence, excessive dependence, fear of abandonment. It is important that the attachment bond develops adequately, since good development of the person derives from this: the attachment model becomes an aspect on which the adult's personality is based and will influence future relationships. [5]. According to Bartholomew and Horowitz (1991) four attachment prototypes have been identified: (a) Secure: derives from a balanced combination of intimacy and autonomy. Subjects confident handle relationships with ease as they have a positive self-image. The securely attached person is generally open-minded and sensitive. (b) Preoccupied: Indicates high levels of concern about relationships. Worried individuals tend to be extremely in need of support and attention; behaviorally and emotionally they are unstable and hypersensitive. Furthermore, they are inclined to devalue themselves and to be excessively dependent on the approval of others, tending to idealize others. (c) Dismissing: indicates the denial of intimacy. Avoidant subjects exaggerately express independence and invulnerability; they have a negative view of others as opposed to a positive perception of themselves. To maintain this positive image they distance themselves emotionally from others and, over time, are led to see themselves as fully autonomous. Therefore, they achieve autonomy and a feeling of self-worth at the expense of intimacy. (d) Fearful: indicates fear of intimacy. Fearful subjects have a negative view of both themselves and others; they crave social contact and intimacy, but distrust others and fear rejection, so they avoid social situations [6, 7].

The onset of a chronic disease can represent a "threat" for the relationship between patient and his/her parental figures, according to the type of bond that already exists. Few studies in the literature have analyzed attachment theory in chronic disease, with particular reference to the role that insecure attachment can play in the management of these conditions, with a negative impact on quality of life and difficulties in psychosocial adaptation. The review by Meredith and Strong (2019) highlighted how further research is needed to better understand the link between insecure attachment and chronic diseases, to develop targeted treatment protocols [1]. Maras et al. (2021) studied attachment in 90 adult subjects with chronic disease and their results showed that avoidant attachment was significantly related to avoidant-type coping style, lower health-related self-efficacy and lower quality of perceived life. The results of this research also suggest that individuals with avoidant attachment require greater attention and care in order to accept their condition and find new ways of adaptation [1, 8]. Jimenez (2017) stressed how attachment theory has an impact on the doctor-patient relationship and on therapeutic adherence. In particular, insecure attachment is associated with poorer therapeutic adherence and a more significant mortality [2]. Brenk-Franz et al. (2017) found, in 209 patients with chronic disease, the mediating role of the doctor-patient relationship between attachment bond and self-efficacy of subjects with multiple chronic diseases [4]. In a study by Agostini et al. (2014) it was found that, out of 103 patients suffering from inflammatory bowel disease, the insecure attachment style prevailed, which was also a significant predictor of a worse quality of life, with particular reference to the dimension of mental health [9]. Agostini et al. (2016) also investigated the relationship between attachment and perceived stress in 101 patients with ulcerative colitis. Insecure attachment was found to be a significant predictor of greater perceived stress [10].

Rückert-Eheberg et al. (2019) investigated the association between the type of anxious and avoidant attachment and suicidal ideation in a sample of 207 patients with chronic disease between 50 and 85 years of age. Anxious attachment, unlike avoidant attachment, was associated with suicidal ideation, present in 13% of patients. In patients with suicidal ideation, 85% presented insecure attachment [3].

Knowing the attachment style of patients with chronic disease can be useful for improving coping skills, which are often lacking, in individuals with dysfunctional attachment styles. The results of a study by Andersen et al. (2019) showed that both anxious and avoidant attachment styles are associated with physical and psychosocial disabilities and also moderate their intensity. Although the effect of this moderation is mild, the authors concluded that attachment represents a valuable variable that can be evaluated in subjects with certain chronic conditions, for the purpose of a more optimal "recovery" [11].

The literature therefore shows that attachment theory is a useful model that can have important clinical implications in the treatment of chronic diseases, just as the identification of the attachment style is important to identify patients at risk and their needs over time, to be able to intervene appropriately.

To the best of our knowledge, there are no studies that examine attachment style in hemodialysis patients. Unlike other chronic diseases, hemodialysis patients find themselves in an unusual existential condition. Hemodialysis patients are under treatment, which is necessary for their survival, but they maintain a condition of physical and mental suffering [12–17]. The international literature confirms that hemodialysis treatment represents a chronic stressful event with clinical, psychological and psychopathological outcomes [18–24].

Research conducted over the last few years on different attachment styles has shown that adults with a secure attachment style show greater resilience to stress, disturbing life events and traumatic experiences than those with insecure attachment. Therefore investigating the relationship between types of attachment and coping with chronic diseases can prove useful as it is possible to identify early subjects with insecure attachment and intervene in a targeted manner.

The aim of our study was to investigate, in a sample of hemodialysis patients, attachment style and parental bond, to evaluate the impact on any psychic symptoms and quality of perceived life.

Particularly, we are interested in investigating how attachment style can influence coping with chronic disease, in line with the recent literature on the topic. In particular, it is hypothesized that a secure attachment style could constitute a protective factor for the mental health and quality of life of dialysis patients, as it would allow the person to better manage the problems and difficulties inherent in this path of illness, on the contrary, an insecure attachment style is hypothesized to be associated with a greater presence of psychopathology and a worse perception of quality of life, with difficulty in coping with this chronic condition.

## Methods

### Participants

The study involved hemodialysis patients who received outpatient hemodialysis therapy in private dialysis centers. The participants were recruited at the Organ Transplant Unit of an Italian University Hospital between September 2021 and February 2022 by telephone contact to update the waiting list for kidney transplantation. After the first telephone interview, one/two face-to-face interviews followed to define patients to be included in the study. The selection was based on the following criteria: having been on hemodialysis treatment for at least 1 year, age greater than or equal to 30. The authors defined as inclusion criteria people aged 30 or over, since the subjects undergoing dialysis in the research reference centers had a higher percentage of age ranging from 30 years upwards, there were very few dialysis patients under the age of 30, so the authors decided to include this age group in the sample.

The exclusion criteria were: Having been on hemodialysis treatment for at least 1 year, age less than 30, having psychiatric disorders or concomitant use of psychiatric drugs that could have influenced cognitive aspects and emotional issues. Subjects were screened by the means of the The Structured Clinical Interview for DSM-5 (SCID-5) in order to exclude any psychiatric disorder. The SCID-5 is a semistructured interview guide for making the major DSM-5 diagnoses. It is administered by a clinician or trained mental health professional who is familiar with the DSM-5 classification and diagnostic criteria; personality disorders were assessed using the Structured Clinical Interview for DSM-5 Personality Disorders (SCID-5-PD), which is also a semi-structured tool [25–27].

The whole sample consisted of 138 hemodialysis patients on the waiting list for kidney transplantation (updated to November, 2021), of which 39 had been temporarily suspended from the list because of failure to update blood chemistry and instrumental tests, another 49 did not participate in the study for the following reasons: 5 deceased, 10 foreigners with difficulties in understanding the Italian language, 15 residing in locations far from the University Hospital and 19 suffering from cancer or COVID-19 infection.

Fifty patients joined the study (of which 31 were on the waiting list suitable for their first transplant, 19 on the list for second transplant after chronic rejection of the first transplant).

The present study was approved by the ethics committee and carried out according to the declaration of Helsinki (World Medical Association, 2013). Prior to inclusion in the study, we received written informed consent from all participants.

### Measures

The tests administered were the following: Attachment Style Questionnaire (ASQ) for the identification of attachment styles, Parental Bonding Instrument (PBI) to evaluate parental bond, Short Form Health Survey-36 (SF-36) for the evaluation of perceived quality of life, Middlesex Hospital Questionnaire (MHQ) for the analysis of main symptoms and relevant traits.

The ASQ is a self-administered questionnaire created by Feeney, Noller and Hanrahan in 1994 [28], which investigates the dimensions of attachment and the differences in styles. It consists of 40 items and uses a 6-point scale (from 1 = totally disagree to 6 = totally agree). It is composed of five scales which are: confidence (F1), discomfort with closeness (F2), need for approval (F3), concern with relationships (F4), and relationships as secondary (F5). According to the four-prototype models proposed by Bartholomew and Horowitz (1991), the five factors correspond to the following attachment styles: secure (F1); avoidant (F2); preoccupied (F3), fearful (F4); and dismissing (F5) [6]. In our study we used the Italian translation by Fossati et al. 2007, approved by the author Judith Feeney. The five scales of the questionnaire showed adequate internal consistency (Cronbach's alpha coefficients between 0.76 and 0.84) [7]. Also, with regard to our sample of hemodialysis patients, the five scales of the ASQ questionnaire showed good reliability (α = 0.85). The PBI by Parker, Tupling and Brown from 1979 is an instrument that measures two distinct dimensions: care and overprotectiveness, both maternal and paternal. It is a self-administered questionnaire, consisting of 25 items (12 for care and 13 for overprotection) on a 4-point Likert scale (from 0 = unlikely to 4 = very likely). The questionnaire aims to evaluate the type of parental attachment observable in the sample and investigates four affective styles: 1. Affectionate constraint: parents with high scores on both the "Care" and "Overprotection" scales; 2. Optimal parenting: parents with a high score on the "Care" scale and low score on the "Overprotection" scale 3. Affectionless control: parents with low score on the "Care" scale and high score on the "Overprotection" scale 4. Neglectful parenting: parents with low scores on both scales. Assignment to “high” or “low” categories is based on the following cut-off scores: For mothers, a care score of 27.0 and a protection score of 13.5. For fathers, a care score of 24.0 and a protection score of 12.5. The PBI has been found to have good reliability and validity based on several studies. In the original study the PBI possessed good internal consistency and re-test reliability [29]. Also, with regard to our sample of hemodialysis patients, the PBI showed good reliability (α = 0.89).

The SF-36 is a questionnaire that allows you to evaluate the general health and emotional state through 36 items. It features eight scaled scores that correspond to the weighted sums of the questions in their section. The eight sections are: vitality (VT), physical functioning (PF), bodily pain (BP), general health perceptions (GH), physical role functioning (PR), emotional role functioning (ER), social role functioning (SR) and mental health (MH). Furthermore, the SF-36 evaluates two global indices related to physical and emotional health: Physical Component Scale (PCS) and Mental Component Scale (MCS). The score on each scale ranges from 0 to 100. The higher the score, the better the perception of a good quality of life. A score of 50 represents the normal reference value for each dimension and for the two global indices. The validity and reliability for SF 36 have been confirmed in patients with chronic kidney disease and in kidney transplant recipients [13, 30, 31]. Also, with regard to our sample of hemodialysis patients, the SF-36 showed good reliability (α = 0.92).

The MHQ is intended to measure the severity of the symptoms or behavior being explored. It examines six specific symptoms, namely: fluctuating anxiety (ANX), phobic anxiety (PHOB), obsessive–compulsive traits (OBS), somatic symptoms (SOM), depressive symptoms (DEP), and hysteria (HY). The MHQ is a questionnaire composed of 48 items, of which a dichotomous part, which is represented by "yes/no, 0/2" answers and another part evaluated on a three-level scale (0–1-2) of frequency (never, sometimes, often). The normal reference values, for each of the traits evaluated, are the following: 5.1 ANX, 2.9 PHOB, 5.8 OBS, 3.2 SOM, 3.3 DEP, and 7.5 HY. The MHQ has been found to be a reliable instrument and also valid as a profile measure [32, 33]. Also, with regard to our sample of hemodialysis patients the MHQ showed good reliability (α = 0.87).

### Data analysis

We ran a set of basic statistical analyses on our data, t for group differences, correlation, and linear regression. Statistical Package for Social Science (SPSS) version no. 27 was used for the analyses. The data satisfied the assumptions of normality of the distribution, suitable for parametric analyses [34].

## Results

The socio-demographic and clinical data of our patients are summarized in Table 1.

**Table 1:** Demographic characteristics of patients included in the study

**Characteristics:**		
**Age, mean ± SD (range)**	**57.82 ± 10.63**	
	**N**	**%**
Male sex	30	60.7%
BMI (Kg/m^2^)	30.1 ± 2.4	
**Education**	**N**	**%**
High school diploma or degree	6	26.45%
Middle school or lower	44	73.55%
**Time spent on dialysis, mean (months) ± SD**		40.92 ± 47.31
**Years of disease, mean ± SD**		19.51 ± 13.54
**Original Nephrological disease**	**N**	**%**
Hypertensive nephrosclerosis	11	21%
Diabetic nephropathy	5	11%
Chronic glomerulonephritis	19	38.57%
Polycystic kidney	13	25.43%
Unknown	2	4%

Regarding the demographic and clinical characteristics of the patients included in the study, the subjects had a mean age of 57.82 years and 60.7% of the subjects were male. Regarding the level of education, 26.45% had high school diploma or degree, 73.55% middle school or lower. Regarding the length of dialysis treatment, the mean was 40.92 months. Regarding the original nephrological disease, 21% had hypertensive nephrosclerosis, 11% diabetic nephropathy, 38.57% chronic glomerulonephritis, 25.43% polycystic kidney and 4% unknown.

Table 2 shows the mean and standard deviation of ASQ, PBI, SF-36, MHQ and demographic characteristics of patients included in the study.

**Table 2:** Mean and standard deviation of ASQ, PBI, SF-36, MHQ and demographic characteristics of patients included in the study

	M	SD
F1	40.68	7.01
F2	40.17	7.86
F3	17.96	6.07
F4	27.36	12.82
F5	16.51	6.29
M care	28.02	7.07
F care	26.22	7.96
M overp	12.44	7.72
F overp	10.40	7.65
VT	61.41	19.48
PF	75.81	22.39
BP	71.02	26.65
GH	55.33	19.48
PR	77.12	33.30
ER	78.37	36.11
SR	84.81	20.47
MH	74.25	16.16
PCS	45.14	8.37
MCS	49.54	9.17
ANX	3.58	3.00
PHOB	3.66	2.23
OBS	4.47	2.38
SOM	4.68	2.93
DEP	3.56	2.24
HY	2.77	2.14
AGE	57.82	10.63
E	10.55	3.19
TD	40.92	43.41
YD	19.51	13.54

*Abbreviations*: *F1* Secure, *F2* Avoidant, *F3* Preoccupied, *F4* Fearful, *F5* Dismissing, *M care*, mother care, *F care* father care, *M overp* mother overprotection, *F overp*, father overprotection, *VT* Vitality, *PF* Physical Functioning, *BP* Bodily Pain, *GH* General Health, *PR* Physical Role Functioning, *ER* Emotional Role Functioning, *SR* Social Role Functioning, *MH* Mental Health, *PCS* Physical Component Scale, *MCS* Mental Component Scale, *ANX* Anxiety, *DEP* Depression, *SOM* Somatization, *PHOB* Phobia, *OBS* Obsession, *HY* Hysteria, *E* Education, *TD* Time spent on dialysis, *YD* Years of disease

The results did not show significant gender differences in ASQ, PBI, SF-36, and MHQ calculated through the t-test of independent samples (Table 3). There were gender differences only in ER of SF-36, in which men showed a better perception of their emotional role than women, and in PHOB of MHQ, in which women showed a greater presence of phobic traits than men. Regarding the ASQ, the following mean scores were found factor F3 25.68 in females, F4 factor 16.39 in males, and 16.68 in females. Regarding the PBI, high mean scores were found in "Mother care" (30.41 in males, 24.55 in females) and "Father care" (26.21 in males, 26.25 in females) of PBI scales (for normal reference values, see “Measures” section). The mean size scores of the SF-36 were adequate; the two general PCS indices (45.54 in males, 44.60 in females) and MCS (47.70 in females) of SF-36 (for normal reference values, see “Measures” section) were slightly below the norm. Study participants showed high mean scores in SOM (4.03 in males, 5.68 in females) and PHOB (3.17 in males, 4.42 in females) of MHQ (for the normal reference values, see “Measures” section).

**Table 3:** Gender differences in ASQ, PBI, SF-36, and MHQ measured using *t*-test of independent samples

	Gender	Mean	SD	t	*p*-value	Mean difference	SE difference	95% CI of the difference	
								Lower	Upper
F1	M	41.79	6.64	1.32	0.862	2.73	2.06	-1.43	6.90
	F	39.05	7.41	1.29			2.11	-1.55	7.02
F2	M	40.39	8.96	0.23	0.162	0.55	2.36	-4.20	5.30
	F	39.84	6.10	0.25			2.19	-3.87	4.97
F3	M	28.50	15.16	0.73	0.562	2.81	3.83	-4.90	10.53
	F	25.68	8.38	0.81			3.45	-4.14	9.77
F4	M	16.39	6.69	-0.15	0.689	-0.29	1.89	-4.09	3.51
	F	16.68	5.81	-0.15			1.83	-4.00	3.41
F5	M	17.64	5.24	-0.83	0.093	-1.51	1.81	-5.16	2.13
	F	19.16	7.18	-0.78			1.92	-5.43	2.40
M care	M	30.41	5.59	3.09	0.102	5.86	1.89	2.05	9.67
	F	24.55	7.68	2.92			2.00	1.77	9.95
F care	M	26.21	8.54	-0.01	0.191	-0.04	2.33	-4.74	4.66
	F	26.25	7.25	-0.01			2.26	-4.61	4.52
M overp	M	9.62	6.53	-3.41	0.419	-6.92	2.03	-11.01	-2.84
	F	16.55	7.61	-3.31			2.09	-11.06	-2.69
F overp	M	7.72	6.76	-3.23	0.801	-6.57	2.03	-10.66	-2.48
	F	14.30	7.31	-3.18			2.06	-10.74	-2.40
VT	M	61.86	17.56	0.22	0.116	1.05	4.66	-8.34	10.45
	F	60.80	13.32	0.23			4.46	-7.92	10.03
PF	M	80.46	36.49	0.37	0.187	11.16	6.42	-1.76	24.08
	F	69.30	29.24	0.38			6.60	-2.21	24.54
BP	M	71.36	24.99	0.10	0.257	0.80	7.88	-15.06	16.68
	F	70.55	29.47	0.10			8.10	-15.62	17.24
GH	M	57.07	21.39	0.72	0.151	4.17	5.73	-7.36	15.71
	F	52.90	16.67	0.75			5.49	-6.90	15.24
PR	M	78.70	36.49	0.37	0.193	3.70	9.92	-16.27	23.68
	F	75.00	29.24	0.38			9.59	-15.62	23.03
ER	M	91.61	23.43	3.30	0.001	31.75	9.60	12.42	51.09
	F	59.85	42.73	3.01			10.53	10.15	53.36
SR	M	85.21	22.57	0.15	0.135	0.96	6.05	-11.23	13.15
	F	84.25	17.67	0.16			5.81	-10.74	12.67
MH	M	76.29	17.03	1.03	0.496	4.88	4.73	-4.63	14.40
	F	71.40	14.81	1.05			4.61	-4.42	14.19
PCS	M	45.54	8.68	0.37	0.574	0.93	2.47	-4.04	5.91
	F	44.60	8.12	0.38			2.44	-4.00	5.87
MCS	M	50.86	9.53	1.18	0.730	3.15	2.67	-2.22	8.54
	F	47.70	8.54	1.20			2.62	-2.22	8.54
ANX	M	2.79	2.30	-2.36	0.073	-1.99	0.84	-3.69	-0.29
	F	4.79	3.56	-2.16			0.92	-3.88	-0.10
PHOB	M	3.17	1.69	-1.94	0.028	-1.24	0.64	-2.53	0.04
	F	4.42	2.75	-1.76			0.70	-2.69	0.20
OBS	M	4.10	2.07	-1.35	0.258	-0.94	0.69	-2.35	0.45
	F	5.05	2.75	-1.28			0.74	-2.46	0.56
SOM	M	4.03	2.63	-1.96	0.146	-1.65	0.84	-3.34	0.04
	F	5.68	3.14	-1.89			0.87	-3.42	0.12
DEP	M	3.17	2.15	-1.50	0.775	-0.98	0.65	-2.30	0.33
	F	4.16	2.31	-1.48			0.66	-2.33	0.36
HY	M	3.00	2.00	0.91	0.794	0.57	0.63	-0.69	1.85
	F	2.42	2.36	0.88			0.65	-0.75	1.91

*Abbreviations*: *M* Male, *F* Female, *ASQ* Attachment Style Questionnaire, *F1* Secure, *F2* Avoidant, *F3* Preoccupied, *F4* Fearful, *F5* Dismissing, *PBI* Parental Bonding Instrument, *M care* mother care, *F care* father care, *M overp* mother overprotection, *F overp* father overprotection, *SF-36* Short Form Health Survey 36, *VT* Vitality, *PF* Physical Functioning, *BP* Bodily Pain, *GH* General Health, *PR* Physical Role Functioning, *ER* Emotional Role Functioning, *SR* Social Role Functioning, *SR* Social Role Functioning, *MH* Mental Health, *PCS* Physical Component Scale, *MCS* Mental Component Scale, *MHQ* Middlesex Hospital Questionnaire, *ANX* Anxiety, *DEP* Depression, *SOM* Somatization, *PHOB* Phobia, *OBS* Obsession, *HY* Hysteria

Pearson's correlations between SF-36, MHQ and demographic characteristics of patients included in the study (age, education, time spent on dialysis and years of disease) are shown in Table 4. Age is negatively correlated with physical activity (PF) of SF-36 (*r* = -0.350; *p* < 0.05). Education is negatively correlated with general health (GH) of SF-36 (*r* = -0.328; *p* < 0.05). Years of disease are negatively correlated with physical pain (BP) (-0.467 *p* < 0.01) and physical health index (PCS).

**Table 4:** Correlations through Pearson’s coefficient (*r*) between SF-36, MHQ and demographic characteristics of patients included in the study

	**Age**	**E**	**TD**	**YD**
VT	-0.075	-0.166	-0.034	0.103
PF	-0.350*	0.064	-0.113	0.013
BP	-0.162	0.029	-0.151	-0.467^**^
GH	-0.068	-0.328*	-0.238	-0.310
PR	0.080	-0.129	-0.148	0.069
ER	0.055	-0.143	-0.020	-0.057
SR	0.262	-0.023	-0.127	0.190
MH	0.068	-0.215	-0.104	0.232
PCS	-0.252	-0.062	-0.166	-0.357*
MCS	0.174	-0.141	-0.041	0.214
ANX	0.026	-0.005	0.213	-0.060
PHOB	0.179	-0.056	-0.185	0.083
OBS	0.066	0.059	0.074	0.069
SOM	0.166	0.041	0.350*	-0.076
DEP	-0.064	0.173	0.126	-0.135
HY	0.096	-0.230	0.141	-0.185

*Abbreviations*: *SF-36* Short Form Health Survey-36, *VT* Vitality, *PF* Physical Functioning, *BP* Bodily Pain, *GH* General Health, *PR* Physical Role Functioning, *ER* Emotional Role Functioning, *SR* Social Role Functioning, *MH* Mental Health, *PCS* Physical Component Scale, *MCS* Mental Component Scale, *MHQ* Middlesex Hospital Questionnaire, *ANX* Anxiety, *DEP* Depression, *SOM* Somatization, *PHOB* Phobia, *OBS* Obsession, *HY* Hysteria, *E* Education, *TD* Time spent on dialysis, *YD* Years of disease

*Significance *p* < 0.05

**Significance *p* < 0.01

(-0.357 *p* < 0.05) of SF-36. Time spent on dialysis is negatively correlated with somatization (SOM) of the MHQ (-0.350 *p* < 0.05). There is no significant correlation between ASQ and PBI with the characteristics of the sample. Pearson's correlations between ASQ, PBI, MHQ and SF-36 are shown in Table 5.

**Table 5:** Correlations through Pearson’s coefficient (*r*) between ASQ, PBI, MHQ and SF-36

	**VT**	**PF**	**BP**	**GH**	**PR**	**ER**	**SR**	**MH**	**PCS**	**MCS**
F1	0.260	0.001	0.060	0.339*	0.038	0.263	0.121	0.547**	-0.025	0.373*
F2	-0.186	0.165	-0.069	-0.225	-0.193	0.081	-0.105	-0.145	-0.112	-0.032
F3	-0.185	-0.067	-0.095	-0.215	0.134	-0.083	-0.113	-0.248	0.010	-0.213
F4	-0.279	-0.062	-0.007	-0.259	0.130	-0.157	-0.095	-0.344*	0.051	-0.299*
F5	-0.047	-0.187	-0.386**	-0.198	-0.067	-0.198	0.125	0.037	-0.272	0.035
M care	0.106	0.054	-0.101	0.037	0.077	0.377**	0.344*	0.250	-0.025	0.373*
F care	0.176	0.163	-0.063	0.250	-0.053	0.069	0.137	0.211	0.043	0.170
M overp	-0.041	0.033	0.231	0.076	0.142	-0.122	-0.022	-0.037	0.215	-0.066
F overp	-0.075	-0.062	0.191	-0.100	0.006	-0.310*	-0.017	-0.131	0.136	-0.205
ANX	-0.281	-0.205	-0.319*	-0.249	-0.151	-0.557**	-0.243	-0.385**	-0.149	-0.421**
PHOB	0.016	0.324*	-0.275	-0.177	-0.122	-0.376*	0.135	-0.007	-0.262	-0.013
OBS	0.105	-0.104	-0.020	-0.130	0.022	-0.016	0.025	-0.019	-0.094	0.099
SOM	-0.504**	-0.283	-0.405**	-0.235	-0.415**	-0.536**	-0.357*	-0.486**	-0.310*	-0.486**
DEP	-0.126	-0.109	-0.061	-0.119	-0.148	-0.388**	-0.319*	-0.333*	0.001	-0.369*
HY	-0.067	-0.114	0.050	0.117	0.019	-0.111	0.075	-0.091	0.071	-0.089

*Abbreviations*: *ASQ* Attachment Style Questionnaire, *F1* Secure, *F2* Avoidant, *F3* Preoccupied, *F4* Fearful; *F5*, Dismissing, *PBI*, Parental Bonding Instrument, *M care* mother care, *F care* father care, *M overp* mother overprotection, *F overp* father overprotection, *M care* Mother care, *F care* Father care, *M overp* Mother overprotection. *F overp* Father overprotection, *ANX* Anxiety, *PHOB* Phobia, *OBS* Obsession, *HY* Hysteria, *SF-36* Short Form (36) Health Survey, *PF* physical functioning, *PR* physical role functioning, *BP* bodily pain, *GH* general health perceptions, *VT* vitality, *SR* social role functioning, *ER* emotional role functioning, *MH* mental health, *PCS* Physical component scale, *MCS* Mental component scale

^*^Significance *p* < 0.05

^**^Significance *p* < 0.01

Secure attachment style (F1) correlated with good general health (GH) (*r* = 0.339; *p* < 0.05), good mental health (MH) (*r* = 0.547; *p* < 0.001) and mental component scale (MCS) (*r* = 0.373; *p* < 0.05). The dismissing attachment style (F5) of the ASQ was significantly correlated with bodily pain (BP) of the SF-36 (*r* = 0.386; *p* < 0.01). The fearful attachment style (F4) was negatively correlated with mental health (MH) (*r* = -0.344; *p* < 0.05) and with mental component scale (MCS) (*r* = -0.299 *p* < 0.05). Maternal care (PBI M Care) was positively correlated with social role functioning (SR) (M Care/SA *r* = 0.344 *p* < 0.05), emotional role (M Care/ER *r* = 0.377 *p* < 0.01) and mental component scale (M Care/MCS *r* = 0.355 *p* < 0.01) of the SF-36. Neither maternal overprotection nor paternal care correlated with the SF-36 dimensions. Paternal overprotection was instead negatively correlated with the emotional role of SF-36 (P Overp/ER *r* = -0.310 *p* < 0.05). The anxiety dimension (ANX) of MHQ negatively correlated with bodily pain (BP) (*r* = -0.319 *p* < 0.05), emotional role limitations (ER) (*r* = -0.557 p < 0.001), mental health (MH) (*r* = -0.385 *p* < 0.001) and the mental component scale (MCS) (*r* = -0.421 *p* < 0.001) of SF-36. The somatizations of the MHQ correlated with all the dimensions of the SF-36, except for physical activity (PF). The phobia dimension of the MHQ negatively correlated with Physical activity (PF) (*r* = -0.324 *p* < 0.05) and emotional role limitations (ER) (*r* = -0.376 *p* < 0.05) of the SF-36.

The results of the multivariate analysis (linear regression) are shown in Table 6.

**Table 6:** Linear Model of Predictors F1, F4, (ASQ) of MH (SF-36) with 95% Bias Corrected and Accelerated Confidence Intervals Reported in Parentheses. Independent variable: F1 and F4 (ASQ); Dependent variable: MH (SF-36)

	**B**	**Std. Error**	**Beta**	**P**
Constant	36.424	19.646	(-3.282 to 76.130)	.071
F1	1.164	.432	.534 (.267 to 2.062)	.013*
F4	.041	.169	.043 (-.310 to .392)	.811

*Abbreviations*: *F1* Secure, *F4* Fearful*, ASQ* Attachment Style Questionnaire, *MH* Mental Health, *SF-36* Short Form Health Survey-36

^*^Significance *p* < 0.05

The authors included as control variables: age, education, time spent on dialysis and years of disease. The independent variable F1 of the ASQ was significantly associated with the dependent variable MH of the SF-36.

## Discussion and Conclusions

Attachment styles constitute specific configurations of the child's emotional behavioral response in relation to parental care modalities [35]. These configurations maintain high stability over time and form the basis that can significantly guide subsequent emotional and social development [36]. The quality of care received during childhood can affect the ability to manage disease as adults. It has been noted that individuals who received adequate care are able to cope with pain and disease in an adequate manner and by expressing empathic abilities. On the other hand, people who received inadequate care in childhood may find it difficult to implement appropriate and effective coping skills [37]. Hamama-Raz et al. (2018) investigated whether attachment patterns moderated the link between coping skills and disease acceptance in 94 kidney transplant recipients. The results of the study showed that, where anxious attachment was present, there were low levels of coping and acceptance of one's condition [37].

The aim of our research was to study attachment style and parental bond in a sample of dialysis patients and to evaluate its impact on quality of life and mental health. The path that leads to dialysis treatment is complex, both in terms of the patient's clinical conditions and in terms of his psychological and emotional state. Therefore, the presence of a multidisciplinary team that guarantees not only clinical but also psychological-psychiatric support is very important, thanks to which well-structured educational paths can be defined in order to prevent, as far as possible, psychopathological problems in this type of patients. Occupational therapy also appears to play an important role in this area [13, 18, 24, 38]. In our sample, the perception of one's physical and emotional health state (perceived quality of life) was not compromised, probably related to the fact that patients on the waiting list are constantly monitored also from a psychiatric and psychological point of view: they carry out an accurate initial psychodiagnostic evaluation for the first insertion onto the waiting list, within the three years on the list there follows an update of the exams including the psychiatric and psychological follow-up; moreover, the patients on the list who request it, have the possibility of receiving psychotherapeutic support. However, it is interesting to note how the MHQ analysis showed high mean scores in the somatization and phobia dimensions, compared to the mean reference values, in addition to the significant correlation that emerged between the months spent on dialysis and somatization traits. This could be linked to the fact that dialysis patients live in a continuous state of alert and apprehension due to the condition of "waiting" for the transplantation organ, a condition that makes the subject more vulnerable and predisposed to somatization. Regarding phobia, in dialysis patients, a sense of compulsion and dependence towards dialysis therapy is often present, in addition to the possible presence of specific fears, such as the fear of contagion of infectious diseases, due to the high risk of transmission. Regarding the assessment of the attachment style, despite the confidence (F1) present in our sample, it is also important to note the presence of preoccupation with relationships (F4), corresponding to the fearful attachment described by Bartholomew. In the “fearful” style the subject avoids involvement with others for fear of being rejected. Low self-confidence and a conflict between desire and fear of intimacy prevail [6]. In our study, a significant correlation emerged between the dismissing attachment (F5) and the perception of bodily pain assessed with the SF-36. The dismissing attachment (F5) is characterized by a positive self-model and a negative other-model. People with dismissing attachment have high self-confidence and, at the same time, tend to devalue relationships by avoiding intimacy. At the behavioral level, this attitude manifests itself in the tendency to maintain an emotional distance towards others. Such negative strategies can lead to poorer pain management. Pain also concerns complex psychological aspects, it is something that takes into consideration emotional, cognitive, affective and stress response phenomena. The perception of pain is "filtered" by the type of attachment. Thus, a dismissing attachment system can exacerbate pain and favor aspects (behaviors, coping strategies, evaluations) in contrast with pain control itself [1].

Another important fact, in line with the literature present on the topic [35–37, 39, 40], concerns the style of secure attachment (F1: confidence), which is correlated with general and mental good health, also showing its predictive value on the latter dimension.

In fact, secure attachment increases resilience and improves mental health. Interactions with available attachment figures confer a sense of security, activate positive emotions (relief, satisfaction, gratitude, love), and provide the psychological resources to deal with problems and adversity [7].

Maternal care was also positively correlated with emotional role functioning, social role functioning and mental component scale.

The maternal care received in infancy is in fact related to positive affectivity, the ability to persist in problem solving situations, greater self-confidence and better social adaptation. Furthermore, mothering is also associated with less dependency and greater competence and ability to resolve conflicts. Individuals who received adequate maternal care in childhood are less likely to experience psychopathological problems. Even in the case of chronic diseases, maternal care is a protective factor for mental health [7].

Conversely, fearful style (F4: preoccupation with relationships) was negatively correlated with mental health.

In our study some limitations must be considered: the small number of participants, the cross-sectional nature of the analysis, the use of self-reporting measures, the lack of adjustment for potential confounding variables and the lack of a control group. Nevertheless, the results of our research confirm the positive role of a secure attachment style and a good parental bond for an adequate perceived quality of life, particularly in relation to the dimensions of mental health. These results may have relevant clinical implications as early identification of hemodialysis patients, awaiting transplant, with dysfunctional attachment styles will make it possible to offer them psychotherapeutic support interventions and drug treatments, if necessary, to improve their ability to accept, adapt and manage the disease and to maintain adequate mental well-being.

## Data Availability

The data that support the findings of this study are available on request from the corresponding author. The data are not publicly available due to privacy or ethical restrictions.

## Abbreviations

CKD: Chronic Kidney Disease
DSM: Diagnostic and Statistical Manual of Mental Disorders
ASQ: Attachment Style Questionnaire
PBI: Parental Bonding Instrument
SF-36: Short Form Health Survey 36
MHQ: Middlesex Hospital Questionnaire
F1: Secure
F2: Avoidant
F3: Preoccupied
F4: Fearful
F5: Dismissing
VT: Vitality
PF: Physical Functioning
BP: Bodily Pain
GH: General Health
PR: Physical Role Functioning
ER: Emotional Role Functioning
SR: Social Role Functioning
MH: Mental Health
PCS: Physical Component Scale
MCS: Mental Component Scale
ANX: Anxiety
PHOB: Phobia
OBS: Obsession
SOM: Somatization
DEP: Depression
HY: Hysteria
SPSS: Statistical Package for the Social Sciences
PBI M Care: Parental Bonding Instrument Maternal Care
PBI P Care: Parental Bonding Instrument Paternal Care
